# Tuning iron spin states in single-atom nanozymes enables efficient peroxidase mimicking†

**DOI:** 10.1039/d2sc05679h

**Published:** 2022-10-26

**Authors:** Xiaoqian Wei, Shaojia Song, Weiyu Song, Yating Wen, Weiqing Xu, Yifeng Chen, Zhichao Wu, Ying Qin, Lei Jiao, Yu Wu, Meng Sha, Jiajia Huang, Xiaoli Cai, Lirong Zheng, Liuyong Hu, Wenling Gu, Miharu Eguchi, Toru Asahi, Yusuke Yamauchi, Chengzhou Zhu

**Affiliations:** Key Laboratory of Pesticide and Chemical Biology of Ministry of Education, College of Chemistry, Central China Normal University Wuhan 430079 PR China czzhu@ccnu.edu.cn; Faculty of Science and Engineering, Waseda University 3-4-1 Okubo, Shinjuku Tokyo 169-8555 Japan; JST-ERATO Yamauchi Materials Space-Tectonics Project, International Center for Materials Nanoarchitechtonics (WPI-MANA), National Institute for Materials Science (NIMS) 1-1 Namiki Tsukuba Ibaraki 305-0044 Japan; State Key Laboratory of Heavy Oil Processing, China University of Petroleum Beijing 102249 P. R. China; Institute of High Energy Physics, Chinese Academy of Sciences Beijing Synchrotron Radiation Facility Beijing 100049 P. R. China; School of Materials Science and Engineering, Wuhan Institute of Technology Wuhan 430205 P. R. China; Australian Institute for Bioengineering and Nanotechnology (AIBN), The University of Queensland Brisbane QLD 4072 Australia y.yamauchi@uq.edu.au

## Abstract

The large-scale application of nanozymes remains a significant challenge owing to their unsatisfactory catalytic performances. Featuring a unique electronic structure and coordination environment, single-atom nanozymes provide great opportunities to vividly mimic the specific metal catalytic center of natural enzymes and achieve superior enzyme-like activity. In this study, the spin state engineering of Fe single-atom nanozymes (FeNC) is employed to enhance their peroxidase-like activity. Pd nanoclusters (Pd_NC_) are introduced into FeNC, whose electron-withdrawing properties rearrange the spin electron occupation in Fe(ii) of FeNC–Pd_NC_ from low spin to medium spin, facilitating the heterolysis of H_2_O_2_ and timely desorption of H_2_O. The spin-rearranged FeNC–Pd_NC_ exhibits greater H_2_O_2_ activation activity and rapid reaction kinetics compared to those of FeNC. As a proof of concept, FeNC–Pd_NC_ is used in the immunosorbent assay for the colorimetric detection of prostate-specific antigen and achieves an ultralow detection limit of 0.38 pg mL^−1^. Our spin-state engineering strategy provides a fundamental understanding of the catalytic mechanism of nanozymes and facilitates the design of advanced enzyme mimics.

## Abstract 2

Spin-state engineering was proposed to enhance the peroxidase-like activity of single-atom nanozymes through the electron-withdrawing properties of Pd nanoclusters, which facilitates the heterolysis process of H_2_O_2_ and the desorption of H_2_O.

## Graphical abstract

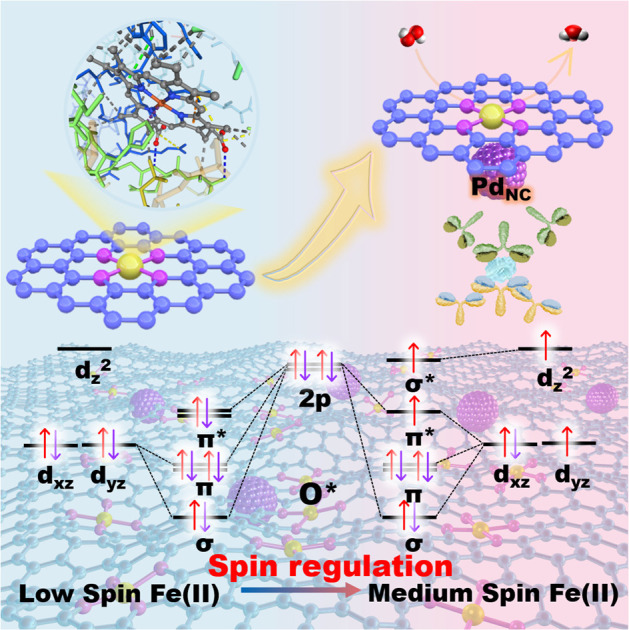

## Introduction

Peroxidases (POD), a family of enzymes that catalytically oxidize certain compounds in the presence of peroxides (H_2_O_2_ in most cases), play a significant role in biological systems, such as H_2_O_2_ detoxification in the human body.^1,2^ They can also be isolated from organisms and applied to various applications such as biosensing, therapy, and environmental protection.^3–5^ However, their large-scale commercialization remains an ongoing issue because of the disadvantages of high cost, difficult recovery and variability.^6^ Nanozymes, nanomaterials with enzyme-like characteristics, have been discovered as substitutes for natural enzymes with the merits of excellent stability, facile storage and easy production.^6–10^ Nevertheless, one obstacle lying in nanozymes emanates from their low catalytic efficiency, which is mainly attributed to their insufficient intrinsic activity.^7^ Overcoming these limitations to acquire highly active POD-like nanozymes is, therefore, a subject of interest and priority.

Compared with simulating functionally, mimicking natural enzymes from the perspective of biological structure may provide a more reasonable strategy to achieve high catalytic performance.^11–14^ Single-atom nanozymes with well-defined electronic and geometric structures are appealing alternatives to natural enzymes as they can mimic the highly evolved catalytic center of natural enzymes at the atomic scale.^12,15,16^ Given the similar heme structure of horseradish peroxidase (HRP), Fe single-atom nanozymes are promising to catalyze H_2_O_2_ activation.^15,17–21^ More importantly, tuning the local environment of single atoms is expected to optimize their electronic structure for enhanced POD-like catalytic performance. For example, heteroatoms such as B, S and P have been introduced as “modulators” to decrease the energy barrier during the reduction of H_2_O_2_.^11,22,23^ FeN_5_, in which the Fe single atom is coordinated by axial N atoms, emulates penta-coordinated heme systems and shows enhanced POD-like performance compared to its FeN_4_ counterpart.^14^ Furthermore, according to the frontier molecular orbital theory, the catalysis process depends on the electron donation and back-donation steps between the reactants/intermediates and metal centers.^24,25^ During the reaction process involving the production of multistep intermediates, the number of unpaired electrons cannot always be conserved, resulting in the spin-related electron transfer and thus the d orbital occupation and spintronic configuration sensitive reaction kinetics and thermodynamics.^26^ Inspired by this, tuning the spin state of metal atoms in single-atom nanozymes is expected to enhance their POD-like activity, realizing the efficient activation of H_2_O_2_ and revealing the underlying relationships between spin states and performance.

In this study, we develop spin-dependent Fe single-atom nanozymes by employing Pd nanoclusters (Pd_NC_) as “modulators” (named FeNC–Pd_NC_) for the first time to achieve enhanced POD-like activity. The synergistic effect between Pd_NC_ and Fe single atoms results in the outstanding POD-like activity of FeNC–Pd_NC_ in comparison to FeNC. Studies show that Pd_NC_ promotes the formation of a greater amount of medium spin (MS) Fe(ii) atoms, which are more active than low spin (LS) Fe(ii) owing to the weakened binding strength towards oxygen-containing intermediates and accelerated H_2_O_2_ activation. The spin-dependent H_2_O_2_ reduction opens an avenue for the rational design of highly active POD mimicking.

## Results and discussion

**Fig. 1:**
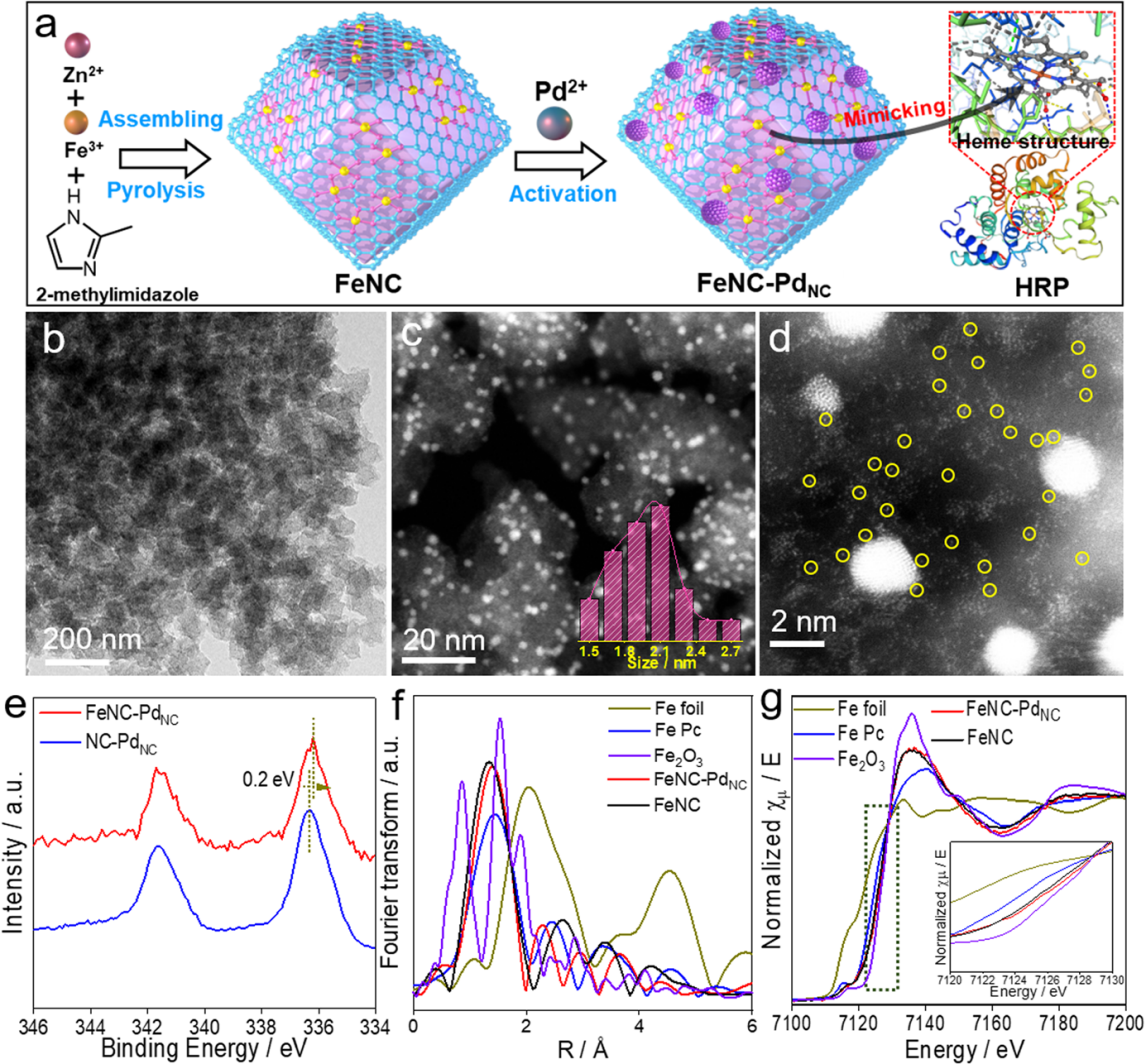
Synthesis and characterization of FeNC–Pd_NC_ nanozymes. (a) Schematic of the synthesis of FeNC–Pd_NC_. (b) TEM, (c) HAADF-STEM and (d) AC-HAADF-STEM images of FeNC–Pd_NC_. Inset in (c) shows the particle size distribution of Pd_NC_. (e) High-resolution Pd 3d XPS spectra of FeNC–Pd_NC_ and NC–Pd_NC_. (f) Fourier-transformed magnitudes of the experimental Fe K-edge EXAFS spectra and (g) XANES spectra of FeNC–Pd_NC_, FeNC, Fe foil, Fe Pc and Fe_2_O_3_. Inset in (g) shows the enlarged XANES spectra.

As displayed in Fig. 1a, FeNC–Pd_NC_ was prepared *via* the confinement effect of metal–organic frameworks (MOFs). Briefly, FeNC was prepared by pyrolysis of Fe-doped zeolitic imidazolate frameworks (ZIF-8) (Fig. S1†). Then, Pd^2+^ ions were incorporated into FeNC through a micropore adsorption strategy and *in situ* reduced to form Pd_NC_.^27^ The rich metal nodes of ZIF-8 and the pores after annealing act as two “claws”, anchoring Fe single atoms and Pd_NC_.^28^ To explore the role of Pd_NC_, NC–Pd_NC_ without Fe single atoms was also prepared using ZIF-8 as the precursor. Scanning electron microscopy (SEM) and transmission electron microscopy (TEM) images of FeNC–Pd_NC_ display its dodecahedral shape inherited from ZIF-8 (Fig. 1b and S2†). The high-angle annular dark-field scanning TEM (HAADF-STEM) image confirms the highly dense and uniformly distributed Pd_NC_ confined in the carbon with an average size of 2.13 nm (Fig. 1c). The Brunauer–Emmett–Teller (BET) area of FeNC–Pd_NC_, FeNC and NC–Pd_NC_ is 547.0, 554.9 and 550.5 m^2^ g^−1^, respectively (Fig. S3 and Table S1†), which guarantees the accessibility of active sites.^29^ The aberration-corrected HAADF-STEM (AC-HAADF-STEM) image shown in Fig. 1d reveals that numerous Fe single atoms surround Pd_NC_. The enlarged AC-HAADF-STEM and corresponding energy dispersive spectroscopy (EDS) mapping confirm the existence of C, N, O, Fe, and Pd elements and the uniformly distributed Pd species in Pd_NC_ (Fig. S4†). X-ray diffraction (XRD) patterns exhibit Pd(111) characteristic peaks in both FeNC–Pd_NC_ and NC–Pd_NC_, confirming the successful introduction of Pd_NC_ into FeNC–Pd_NC_ (Fig. S5†). Inductively coupled plasma optical emission spectrometry (ICP-OES) analysis shows that the contents of Pd and Fe in FeNC–Pd_NC_ were ∼5.6 and ∼0.36 wt%, respectively. Raman spectra (Fig. S6†) indicate that both FeNC–Pd_NC_ and FeNC have rich disorientated graphene structures with a high ratio of D and G band intensities (*I*_D_/*I*_G_).^30^

X-ray photoelectron spectroscopy (XPS) analysis was performed to understand the chemical states of various elements in the nanozymes. The C 1s spectra shown in Fig. S7a† exhibit four peaks corresponding to sp^2^-C, sp^3^-C, C–N_*x*,_ and C=O/C–O.^31^ The N 1s spectra can be deconvoluted into four peaks at 398.5, 399.5, 400.4, and 401.1 eV, which are attributed to pyridinic N, FeN, pyrrolic N, and graphitic N, respectively (Fig. S7b†).^32^ The high ratios of FeN content in FeNC–Pd_NC_ (23.1%) and FeNC (21.6%) indicate the presence of numerous Fe single-atom species (Table S2†). In particular, the binding energy of Fe 2p in FeNC–Pd_NC_ is higher than that in FeNC, whereas Pd shows a decreased binding energy compared to NC–Pd_NC_ (Fig. 1e and S8†). This suggests that electron transfer occurs from Fe single atoms to Pd_NC_, owing to the difference in the electronegativities of Pd and Fe^2+^.^33^ Moreover, the decreased electron density on the Fe center in FeNC–Pd_NC_ may significantly regulate its catalytic behavior.

To further elucidate the changes in the local chemical configuration of Fe species after the introduction of Pd_NC_, the Fe K-edge X-ray absorption fine structure (XAFS) was obtained. As depicted in Fig. 1f, the Fe K-edge extended-XAFS (EXAFS) Fourier-transformed magnitudes of FeNC–Pd_NC_ and FeNC show major peaks at ∼1.4 Å, which is consistent with that of iron-porphyrin (Fe Pc) and attributed to the backscattering between Fe and N atoms.^11,34^ The FeN coordination numbers in FeNC–Pd_NC_ and FeNC obtained *via* the quantitative EXAFS fitting analysis were 4.1 and 4.2, respectively (Fig. S9 and Table S3†). X-ray absorption near edges structure (XANES) spectra were obtained to identify the valence state of Fe species. As displayed in Fig. 1g, the absorption edges of Fe K-edge XANES curves of FeNC–Pd_NC_ and FeNC are between Fe Pc and Fe_2_O_3_. In addition, the valence state of FeNC–Pd_NC_ is more positive than that of FeNC, which indicates the decreased electron density and is consistent with the XPS results.^35,36^ First derivative XANES spectra were obtained to reveal the oxidation state of Fe (Fig. S10†). Overall, the characteristic Fe^2+^ peak (∼7122.6 eV), similar to that of Fe Pc, appears for both FeNC–Pd_NC_ and FeNC, while the peak of FeNC–Pd_NC_ shows a slight positive shift compared to that of FeNC, indicating its relatively positive charge upon the integration of Pd_NC_.

**Fig. 2:**
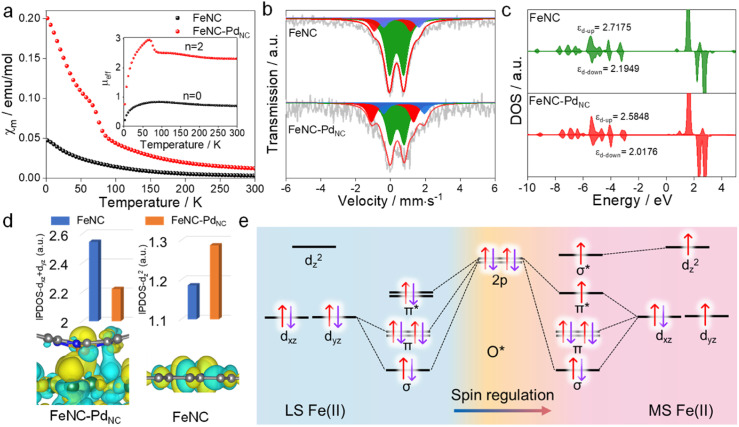
Spin regulation of Fe single atoms. (a) *χ*_m_ plots and the *μ*_eff_, (b) ^57^Fe Mössbauer transmission spectra of FeNC and FeNC–Pd_NC_ and their deconvolution. (c) Projected DOS diagrams of FeNC and FeNC–Pd_NC_. (d) Integrated crystal orbital Hamilton population values of different orbitals of FeNC and FeNC–Pd_NC_ (top), and the HOMO of Fe(ii) in FeNC–Pd_NC_ and FeNC (bottom). (e) Orbital interaction between LS and MS Fe(ii) single atoms and *O adsorbates.

The charge redistribution primarily dominates the electronic configuration of Fe(ii), *i.e.*, the spin state, and thus regulates the catalytic activity. N coordinated Fe(ii) exhibits multiple states, such as LS (d_xy_2 d_xz_2 d_yz_2), MS (d_xy_2 d_xz_2 d_yz_1 d_z^2^_1) and high spin (HS; d_xy_2 d_xz_1 d_yz_1 d_z^2^_1 d_x^2^−y^2^_1) owing to the variation of the coordination environment (Fig. S11†).^37^ In the case of LS Fe(ii), the Fe atom splits into fully occupied low-level orbitals whereas in MS Fe(ii), one electron from the d_yz_ orbital will hop to the d_*z*^2^_ orbital, resulting in unpaired electrons. To verify the spin redistribution experimentally, the zero-field cooling temperature-dependent (ZFC-T) magnetic susceptibility (*χ*_m_) study was carried out. As shown in Fig. 2a, the total effective magnetic moment (*μ*_eff_) was obtained according to the equation, 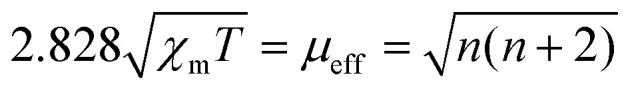 to calculate the number of single spin electrons (*n*).^38^ The *n* of Fe(ii) increases from ∼0.52 in FeNC to ∼2.18 in FeNC–Pd_NC_, confirming the increase in spin state.

For quantification, Mössbauer spectroscopy analysis, which is a powerful tool for identifying the spin polarization configuration, was conducted. Three different doublets (D1–D3) are fitted for FeNC–Pd_NC_ and FeNC, corresponding to LS Fe(ii), MS Fe(ii), and HS Fe(ii), respectively.^39^ FeNC–Pd_NC_ possesses a higher content of MS Fe(ii) (33.4%) than FeNC (17.1%) (Fig. 2b and Table S4†). This could be attributed to the strong interactions between the Fe single atoms and Pd_NC_ in FeNC–Pd_NC_ that effectively reshaped the electronic structure of Fe, achieving the Fe 3d electron-spin configuration transition from LS to MS. Based on this, the electronic spin information of Fe single atoms was further investigated through density functional theory (DFT) calculations. FeN_4_ embedded in graphene was used to simulate FeNC, while the FeNC coupling with Pd (111) facets was used to simulate FeNC–Pd_NC_. As shown in Fig. 2c, the projected density of states (DOS) diagrams reveal that the distance between the d-band center of spin up and spin down (|*ε*_d-up_-*ε*_d-down_|) increases with the introduction of Pd_NC_, indicating a higher spin state for the Fe center. The integral projected density of states analysis of Fe 3d orbital splitting reveals that the increasing spin state mainly originated from the increased Fe d_*z*^2^_ contributions (Fig. 2d), which is also visualized by the spin density diagram (Fig. S12†). Compared to FeNC, the highest occupied molecular orbital (HOMO) of Fe(ii) in FeNC–Pd_NC_ shows one d_z^2^_ orbital perpendicular to the plane within the introduction of Pd_NC_ compared to FeNC (Fig. 2d below).

Typically, Fe-based catalysts strongly bind to oxygen, circumscribing proton–electron transfer and catalytic activity. During the H_2_O_2_ reduction procedure, the 2p orbital of O in H_2_O_2_/intermediates and 3d orbital of Fe sites hybridize, making the 3d electronic state act as spin-dependent gates to regulate electron transfer and orbital interaction in the catalytic reaction.^24^ To confirm this phenomenon, the H_2_O_2_ reduction process in both LS and MS Fe(ii) was theoretically predicted. Due to the scaling relationships of different intermediates, O* is chosen as the model to study its interaction with Fe sites.^40^ Besides, the interaction between Fe-d_xy_/d_x^2^−y^2^_ orbitals and adsorbed O* is negligible because they cannot mix based on symmetry conservation.^41^ As shown in Fig. 2e, the interaction between the Fe center and adsorbed O* changes along with the change in the spin state of Fe(ii). In particular, a half-filled d_z^2^_ orbital in MS Fe(ii) can accept fewer valence electrons from *O with the generation of one σ* orbital, favorable for the desorption of H_2_O_2_/intermediates to trigger a catalytic cycle (Fig. S13†). In this case, the local microenvironment disturbance is exerted to modulate the electronic hopping and occupation of the 3d orbital, achieving the spin reconfiguration and regulating the enzyme-like activity.

**Fig. 3:**
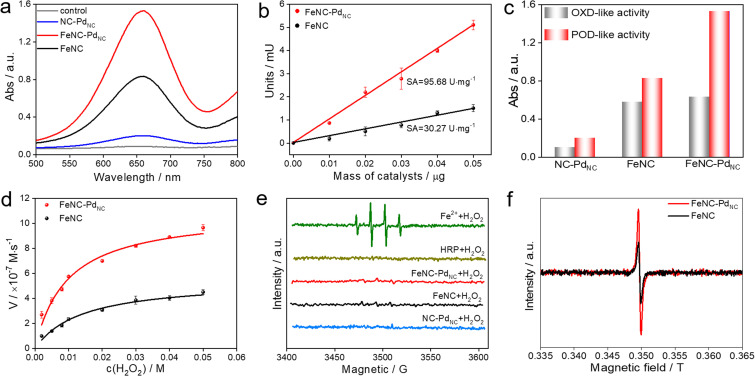
POD-like performances of nanozymes. (a) UV-Vis spectra of FeNC–Pd_NC_, FeNC, and NC–Pd_NC_ in the TMB/H_2_O_2_ system. (b) SA of FeNC–Pd_NC_ and FeNC. (c) Absorbance value of ox-TMB solutions at 652 nm in OXD-like or POD-like reaction systems using FeNC–Pd_NC_, FeNC, and NC–Pd_NC_. (d) Michaelis–Menten kinetic analysis of FeNC–Pd_NC_ and FeNC using different concentrations of H_2_O_2_. (e) EPR spectra of ˙OH produced by different systems. (f) EPR spectra of FeNC and FeNC–Pd_NC_ in the presence of excess H_2_O_2_ at 77 K.

The POD-like catalytic activity of FeNC–Pd_NC_ was quantitively assessed using a chromogenic biochemical reaction of 3,3′,5,5′-tetramethylbenzidine (TMB), which can be oxidized by H_2_O_2_ to ox-TMB in blue color (at 652 nm).^42,43^ As expected, FeNC–Pd_NC_ exhibits two times the catalytic activity of FeNC (Fig. 3a). Besides, the specific activity (SA) value of FeNC–Pd_NC_ was 95.68 U mg^−1^, which is more than three-fold higher than that of FeNC (30.27 U mg^−1^) (Fig. 3b). Meanwhile, the injection of SCN^−^ prominently impairs the POD-like performance of FeNC–Pd_NC_, confirming the main role of Fe single atoms in the POD-like property (Fig. S14†).^13^ Thus, the above results illustrate that Fe single atoms play a critical role in POD-like activity and the existence of Pd_NC_ can significantly boost their activity, despite the sluggish kinetics of H_2_O_2_ reduction at Pd_NC_. Generally, the unsatisfactory specificity of nanomaterials has been a major constraint in the development of nanozymes.^44^ Therefore, the oxidase (OXD)-like activity, the major interfering reaction to POD-like activity was also evaluated (Fig. 3c and S15†). Unlike the POD-like activity of FeNC–Pd_NC_, the OXD-like activity shows negligible enhancement after the integration of Pd_NC_. Moreover, the POD-like activity of FeNC–Pd_NC_ is much higher than that of the addition of FeNC and NC–Pd_NC_ activities, which demonstrates the synergistic effect between Pd_NC_ and Fe single atoms. The Michaelis–Menten equation was applied to assess the POD-like activity of nanozymes (Fig. 3d, S16 and S17†). The Michaelis–Menten constant (*K*_m_), maximum velocity (*V*_max_), and *K*_cat_ (*K*_cat_ = *V*_max_/[S]) are listed in Table S5†. FeNC–Pd_NC_ exhibits higher *K*_cat_ (5.10 s^−1^) than FeNC (2.56 s^−1^) towards H_2_O_2_, indicating its higher POD-like activity. More importantly, FeNC–Pd_NC_ also has competitive catalytic activity compared with other reported nanozymes. Both FeNC–Pd_NC_ and FeNC exhibit high pH-dependent properties similar to natural HRP and higher temperature tolerance than HRP (Fig. S18†). The as-prepared nanozymes maintain their POD-like activity after long-term storage and the treatment of strong acidic and alkaline solutions compared to HRP, suggesting that they can perfectly surmount the disadvantage of the inferior stability of natural enzymes (Fig. S19 and S20†). Therefore, FeNC–Pd_NC_ is a promising alternative to natural enzymes.

To understand the potential catalytic intermediates, electron paramagnetic resonance (EPR) spectroscopy was performed by using 5,5-dimethyl-1-pyrroline *N*-oxide (DMPO) as an ˙OH trapping agent. No signal of ˙OH was detected in HRP/H_2_O_2_ and all nanozymes/H_2_O_2_ systems except for the Fe^2+^/H_2_O_2_ system (Fig. 3e). The existence of ˙OH radical scavenger *t*-butyl alcohol has little effect on the activity of FeNC–Pd_NC_ and FeNC, indicating that ˙OH is not a critical active intermediate in the catalytic reaction (Fig. S21†). Inspired by the catalytic mechanism of natural HRP, the Fe(iv)=O intermediate is considered as the active intermediate.^45,46^ Then a low-temperature (77 K) EPR experiment was performed. As expected, rhombic signals at *g* = 2 were detected after the reaction of FeNC–Pd_NC_ and FeNC with excess H_2_O_2_, suggesting the formation of compound oxoferryl Fe(iv)=O and the porphyrin cation radical (named as the oxo-iron porphyrin π-cation radical [Fe(iv)=O Por^+˙^]) during the reaction process (Fig. 3f).^47^ Notably, the intensity of FeNC–Pd_NC_ is higher than that of FeNC. Considering the heme-like structure of FeNC similar to natural HRP, the essential active intermediate Fe(iv)=O might have formed *via* the bound reactive oxygen species (ROS) pathway while Pd_NC_ is expected to enhance the production of Fe(iv)=O species.

**Fig. 4:**
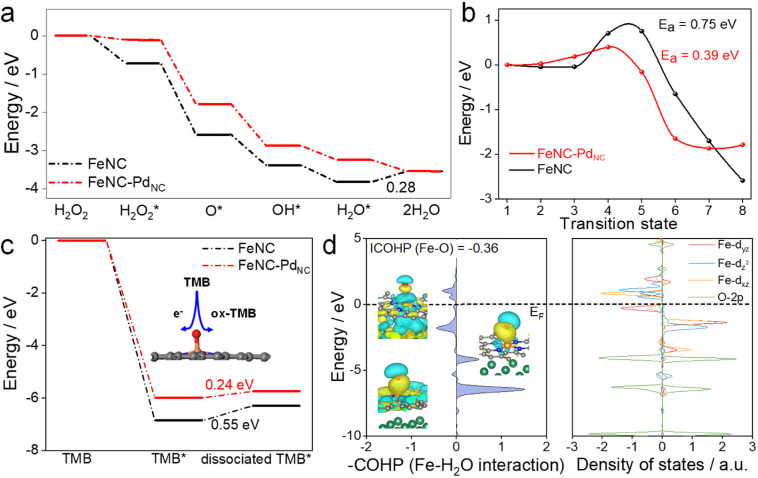
Proposed POD-like mechanism. (a) Free-energy diagram of the POD-like mechanisms of FeNC and FeNC–Pd_NC_. (b) Calculated H_2_O_2_ dehydration process on FeNC–Pd_NC_ and FeNC. (c) Free-energy diagrams of TMB oxidation. (d) Calculated pCOHP and DOS diagram of the Fe–H_2_O* interaction on FeNC–Pd_NC_.

DFT calculations were performed to theoretically understand the effect of the spin state on the POD-like activity. As shown in Fig. 4a and S22,† the H_2_O_2_ heterolytic dissociation route is energetically more favorable for FeNC and FeNC–Pd_NC_. Moreover, the H_2_O* desorption on FeNC is the rate-determining step (RDS) with an energy barrier of 0.28 eV due to the strong binding between Fe single atoms and oxygen-containing species. Encouragingly, this step is exothermic for FeNC–Pd_NC_, indicating that Pd_NC_ integration facilitates the timely H_2_O desorption. Fe(iv)=O, a critical intermediate for TMB oxidation similar to that observed for natural HRP, is produced by the dehydration of the adsorbed H_2_O_2_ as follows: H_2_O_2_ → O* + H_2_O.^48^ As shown in Fig. 4b, the H_2_O_2_ dehydration process is not thermodynamically favorable for FeNC with an energy barrier of 0.75 eV. After the integration of Pd_NC_, this energy barrier is decreased to 0.36 eV on FeNC–Pd_NC_, promoting the formation of Fe(iv)=O intermediate sites. As mentioned previously, the spin state of Fe(ii) fluctuates the interaction between Fe sites and *O, which further improves its oxidation capacity towards TMB. Next, the effect of Pd_NC_ integration on TMB oxidation was evaluated. The oxidation of TMB proceeded through the N–H bond cleavage of TMB and transformation H to Fe(iv)=O along with transfer of an electron from the substrate to the nanozymes. As a result, the dissociation energy of TMB decreases from 0.55 eV in FeNC to 0.24 eV in FeNC–Pd_NC_, thus rendering it more favorable to oxidize TMB (Fig. 4c).

The orbital interactions between H_2_O* and Fe sites were explored. The COHP and DOS analysis reveals that the energy level of the bonding molecular orbital of FeNC–Pd_NC_ shifts to lower energy, whereas that of the antibonding molecular orbital shifts to higher energy, which weakens the Fe–O bond strength in the H_2_O* adsorption configuration (Fig. 4d and S23†). Furthermore, an axial σ* antibonding molecular orbital is observed for H_2_O* on FeNC–Pd_NC_ owing to the electrons in the higher level d_z^2^_ orbital, while the antibonding contribution is mainly from π* antibonding orbitals for FeNC. This unique σ* antibonding orbital contributes to the timely H_2_O desorption from FeNC–Pd_NC_. These DFT results confirm that FeNC–Pd_NC_ with a higher ratio of MS Fe(ii) exhibits highly efficient POD-like activity.

**Fig. 5:**
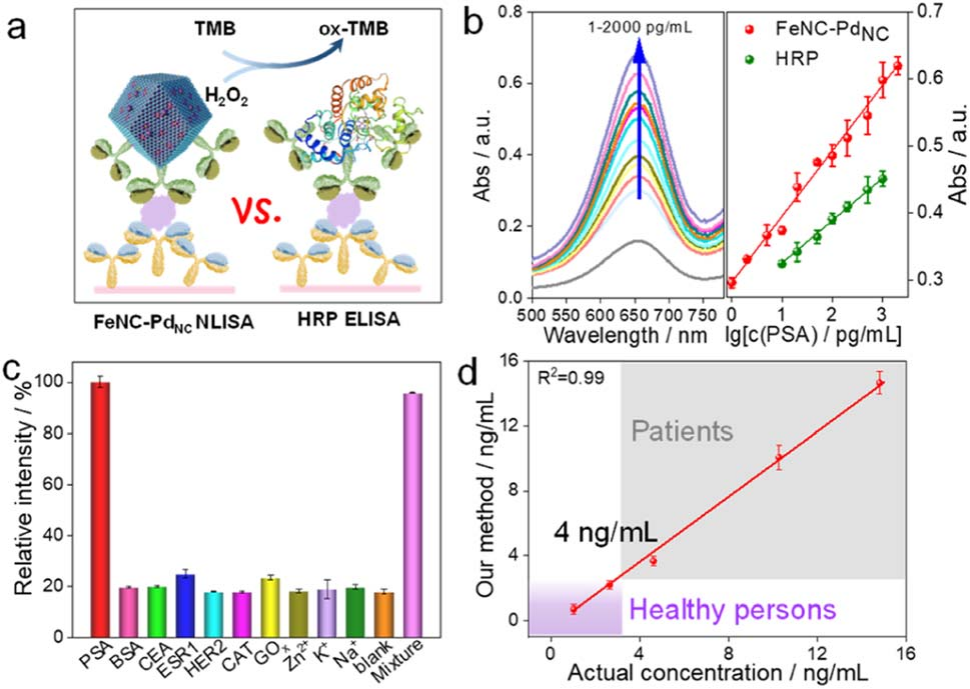
Application of the POD mimics. (a) Schematic illustration of colorimetric detection of PSA. (b) Absorption spectra of ox-TMB with different PSA concentrations (left) and standard curves of PSA detection by using FeNC–Pd_NC_ and HRP as labels (right). (c) Absorbance values for detection of different targets. (d) A linear relationship between the results of our method and the chemiluminescence for the detection of human serum samples.

Subsequently, we performed a proof-of-principle demonstration. FeNC–Pd_NC_ was applied as a label in the colorimetric nanozyme-linked immunosorbent assay (NLISA), and prostate-specific antigen (PSA) was chosen as the model analyte (Fig. 5a).^49–51^ Typically, the surface of FeNC–Pd_NC_ is positively charged, which can conjugate with the labeling antibody of PSA (Ab_2_) as a signal tag through electrostatic interaction (Fig. S24†). The red fluorescence emission in the confocal laser scanning microscope demonstrates the successful immobilization of Ab_2_ (labeled by rhodamine B) on FeNC–Pd_NC_ (Fig. S25†). The absorption spectra and corresponding calibration curves were obtained, where FeNC–Pd_NC_ NLISA exhibits a wide detection range of 1–2000 pg mL^−1^ with a lower variable coefficient of 0.18–5.18% (Fig. 5b and S26†). The limit of detection (LOD) defined by the 3SD method is 0.38 pg mL^−1^. In contrast, the LOD of the traditional HRP-based enzyme-linked immunosorbent assay (ELISA) is 5.38 pg mL^−1^ with a detection range of 10–1000 pg mL^−1^ (Fig. S27†). The sensitivity of FeNC–Pd_NC_ towards PSA is also higher than those in previous reports, suggesting that FeNC–Pd_NC_ has potential immunoassay applications (Table S6†).

In terms of specificity, an obvious characteristic intensity was observed for PSA, while there are negligible changes in the signal intensity for other biomarkers and metal ions (BSA, CEA, ESR1, HER2, CAT, GO_*x*_, Zn^2+^, K^+^, and Na^+^) compared to the blank samples (Fig. 5c). In the presence of all of the above interferents, the FeNC–Pd_NC_ NLISA maintains its high-intensity signal for PSA, indicating its good selectivity and anti-interference ability. Furthermore, FeNC–Pd_NC_ NLISA exhibits outstanding repeatability and reproducibility with low calculated relative standard deviations (RSD; 4.23% and 1.92%, respectively) (Fig. S28†). Stability tests were conducted for ten days, where the RSD was 3.29% (Fig. S29†). By taking advantage of its high activity, selectivity, and stability, FeNC–Pd_NC_ NLISA was used to detect PSA in real serum samples. As expected, the results of FeNC–Pd_NC_ NLISA are consistent with those of chemiluminescence analysis, indicating its remarkable feasibility and accuracy for practical application in the proposed assay (Fig. 5d). Thus, FeNC–Pd_NC_ NLISA has potential applications in clinical diagnosis and can be extended for the detection of other biomarkers.

## Conclusions

Spin-dependent Fe single-atom nanozymes have been developed to achieve enhanced POD-like activity. Experimental and theoretical calculations reveal that the electron-withdrawing effect of Pd_NC_ induces the LS to MS transition of the Fe single atom with the increased electron occupation in the d_z^2^_ orbital. The charge transfer and orbital interactions between the Fe sites and intermediates display spin-dependent features, where the higher electron occupation in the d_z^2^_ is beneficial for the generation of the essential Fe(iv)=O intermediates and promotes the timely desorption of H_2_O*. Thanks to the superior catalytic activity, FeNC–Pd_NC_ can be used to construct NLISA for sensitive detection of PSA, exhibiting satisfactory selectivity and sensitivity. This spin-state engineering strategy for the design of nanozymes not only provides a fundamental understanding of their catalytic mechanism but also promotes the design and development of enzyme-like catalysts for the future.

## Data availability

The data supporting the findings of this study are available within the article and in the ESI.†

## Author contributions

C. Z. and Y. Y. conceptualized and supervised this study. X. W. designed the experiments and wrote the paper. S. S. and W. S. performed the theoretical calculations. Y. W., W. X., Y. C., Z. W., Y. Q., Y. W., M. S., J. H., and X. C. performed the experiments and reviewed the manuscript. L. Z. conducted X-ray absorption experiments and L. J. contributed to XAS data analysis. L. H., W. G., and M. E. provided helpful discussions. All authors discussed and revised the manuscript.

## Conflicts of interest

There are no conflicts of interest to declare.

## Supplementary Material

SC-013-D2SC05679H-s001
