# Supplementary material for: Tuning iron spin states in single-atom nanozymes enables efficient peroxidase mimicking
Source: Chem Sci. 2022 Oct 26;13(45):13574–81. doi: 10.1039/d2sc05679h (PMC9682990; doi:10.1039/d2sc05679h)
Supplement: SC-013-D2SC05679H-s001 [file SC-013-D2SC05679H-s001.pdf]

Electronic Supplementary Material (ESI) for Chemical Science.
This journal is © The Royal Society of Chemistry 2022

Supplementary Methods

Materials.

2-Methylimidazole, zinc nitrate hexahydrate ($Zn(NO_3)_2·6H_2O$), ferric nitrate nonahydrate ($Fe(NO_3)_3·9H_2O$), sodium tetrachloropalladate(II) (Na_2PdCl_4) and $HClO_4$ were purchased from Shanghai Aladdin Bio-Chem Technology Co., Ltd. 3,3′,5,5′-Tetramethylbenzidine (TMB), hydrogen peroxide (H_2O_2), acetic acid (HAc), and sodium acetate (NaAc) were purchased from Sinopharm Chemical Reagent Co., Ltd. Bovine serum albumin (BSA), horseradish peroxidase (HRP), glucose oxidase (GO_x), catalase (CAT) and 5,5-dimethyl-1-pyrroline 1-oxide (DMPO) were purchased from Sigma-Aldrich. All other reagents were analytical-reagent grade and used without any pretreatment. Phosphate buffered saline (PBS) was purchased from Boster Biological Technology Co., Ltd. The antibodies and antigens of prostate-specific antigen (PSA), carcinoembryonic antigen (CEA), estrogen receptor alpha (ESR1), and human epidermal growth factor receptor-2 (HER2) were purchased from Sangon Biotech (Shanghai) Co., Ltd. An enzyme-labeled antibody of PSA was purchased from Shanghai Linc-Bio Co., Ltd. Other chemical reagents were purchased from Sinopharm Chemical Reagent Co., Let (Shanghai, China). The water used for all experiments was prepared using a three-stage Millipore Milli-Q plus 185 purification system and had a resistivity higher than 18.2 MΩ cm.

Synthesis of FeNC. 2-Methylimidazole (3.94 g) was dissolved in 300 mL of methanol with vigorous stirring. Then a 300 mL methanol solution containing 3.39 g $Zn(NO_3)_2·6H_2O$ and 200 mg $Fe(NO_3)_3·9H_2O$ was added to the reaction mixture. The resultant solution was heated at 60 °C for 24 h. The precipitant was centrifuged, thoroughly washed with ethanol for 3 times to remove unreacted linkers, and dried at 60 °C in a vacuum oven to obtain Fe/ZIF-8 crystals. Then, FeNC was prepared by pyrolyzing Fe/ZIF-8 crystals in a tube furnace for 2 h at 900 °C with a heating rate of 5 °C/min under N_2 flow. No additional acidic leaching was performed.

Synthesis of FeNC-Pd$_{NC}$. FeNC (40 mg) was dispersed in 50 mL of water and vigorously sonicated for 10 min. Subsequently, 0.76 mL of Na_2PdCl_4 solution (19.7 mM) was added dropwise to the above suspension while stirring vigorously. For sufficient Pd impregnation, the solution was stirred continuously for 24 h. Then, the resultant mixture was washed with water for 3 times and dried at 60 °C in a vacuum oven. FeNC with its adsorbed Pd was maintained under 5% H_2 gas flow at 400 °C with heating rate of 5 °C/min for 2 h to obtain the final FeNC-Pd$_{NC}$. The mass fraction of Pd in FeNC-Pd$_{NC}$ was 4%.

Synthesis of NC-Pd$_{NC}$. The procedure was similar to that of FeNC-Pd$_{NC}$, except for the addition of $Fe(NO_3)_3·9H_2O$ during the synthesis of FeNC.

Characterization.

The crystal structures of nanozymes were examined by D8 ADVANCE instrument (Bruker, Germany) at 40 kV and 40 mA with Cu Kα radiation (λ = 0.15406 nm) in the 2θ range of 10–80°. The morphologies were characterized by scanning electron microscopy (SEM; Quanta FEG250 field-emission environmental SEM, FEI, USA) and transmission electron microscopy (TEM; JEM-2100 Electron Microscope, Japan). The aberration-corrected high-angle annular dark-field scanning TEM (AC-HAADF STEM) was performed using Titan G2-600 (FEI, USA). The elemental compositions were analyzed by inductively coupled plasma optical emission spectrometry (ICP-OES; Agilent 8800). Raman spectroscopy was performed in backscattering mode using DXR (Thermo Fisher Scientific, USA) with the 780 nm laser. X-ray photoelectron spectroscopy (XPS) measurements were performed by Thermo ESCALAB 250XI (Thermo Fisher, USA) and all of the binding energy data were calibrated by C 1s (284.6 eV). Brunauer–Emmett–Teller (BET) surface are was measured using N_2 adsorption/desorption at 77 K on a MICROMERITICS ASAP 2460 (USA). The Fe oxidation state and coordination structure in the nanozymes and reference compounds were investigated using X-ray absorption spectroscopic methods—X-ray absorption near-edge structure (XANES) and extended X-ray absorption fine structure (EXAFS). These were obtained at 1W1B station in Beijing Synchrotron Radiation Facility (BSRF). The Mössbauer measurements were performed using Wissel MS-500 (MossWinn, Germany) in transmission geometry with constant acceleration mode and $^{57}Co(Rh)$ source with an activity of 25 mCi. The values of isomer shift (IS), electric quadrupole splitting (QS), and relative area of Fe ions were fitted through the least squares method. For zero-field cooling temperature-dependent (ZFC-T) magnetic susceptibility measurements, samples were demagnetized at 293 K by setting an initial field of 50 kOe and decreasing the field stepwise to zero by oscillating at 200 Oe/s. Samples were cooled down to 2 K via a cryocooler-based cooling system at zero fields. Then an external field of 300 Oe was applied and the samples were heated to 300 K at 2.5 K/min. Electron paramagnetic resonance (EPR) spectra were from Bruker A300. All UV-vis spectra were recorded on a multi-mode reader (Tecan Spark, Switzerland).

Evaluation of peroxidase-like activity of the nanozymes

The peroxidase (POD)-like activity was confirmed by the color change of TMB, i.e., the absorbance at 652 nm. The sample was prepared by adding 10 μL of 1 mg mL^{-1} FeNC-Pd_{NC}, FeNC, or NC-Pd_{NC} to a solution of 150 μL of 0.1 M HAc-NaAc buffer (pH 3.5), 100 μL of 100 mM H_2O_2, and 50 μL of 1mM TMB. After 5 min of the reaction, their absorbances were recorded on a multimode reader.

Kinetics assay

The kinetics data were detected by the change in concentration of H_2O_2 or TMB. Typically, 10 µL of FeNC-Pd_{NC}, FeNC, or NC-Pd_{NC} were added into a mixture of 50 µL of 10 mM TMB, 150 µL of 0.1 M Hac-NaAc (pH 3.5), and 100 µL of different concentrations of H_2O_2. The kinetic data were calculated by a typical Michaelis–Menten curve using $v = V_{max}[S]/(K_m+[S])$, where v is the initial velocity, [S] is the concentration of the substrate, K_m is the Michaelis–Menten constant, and V_{max} is the maximal reaction velocity.

Specific activity measurements

The specific activities (SA) of FeNC-Pd_{NC} and FeNC were confirmed by the color change of TMB, which is indicated by the absorbance at 652 nm. Various concentrations of FeNC-Pd_{NC} and FeNC were added into a mixture of 100 µL of H_2O_2 (10 M), 150 µL of HAc-NaAc (0.1 M, pH 3.5), and 50 µL of TMB (100 mM). The SA was calculated by the following equation:

$$SA = \frac{V/(\varepsilon \times l) \times (\Delta A/\Delta t)}{m} \quad (1)$$

where V is the total volume of the reaction solution (µL); ε is the molar absorption coefficient of the colorimetric substrate, which is typically maximized at 39,000 $M^{-1}cm^{-1}$ and 652 nm for TMB; l is the path length of light traveling in the cuvette (cm); A is the absorbance after subtracting the blank value, and ΔA/Δt is the initial rate of change in absorbance at 652 nm min^{-1}; and m is the weight (mg) of the nanozymes in each assay.

Density functional theory calculations

All spin-polarized calculations were performed using Vienna ab initio Simulation Package.[1-2] The Perdew–Burke–Enrzerhof parametrization of the generalized gradient approximation was used to describe the electronic exchange-correlation energies. The criterion of total energy convergence in the optimization of the atomic structure is 10^{-5} eV, the convergence criterion of the atomic force is 0.05 eV/Å, and the cut-off energy of plane wave expansion is 400 eV.[3] The Brillouin zone was sampled using the Monkhorst–Pack method with 2×2×1 k-point meshes. A vacuum layer of 15 Å was used along the c direction normal to the surface to avoid periodic interaction. The climbing image nudged elastic band method was applied for transition state optimizations.

A single FeN_4 moiety was embedded into a single layer graphene to simulate the FeNC. For the calculations of the FeNC-Pd_{NC} composite model, the optimized FeNC model was placed on the five-layer Pd(111) slab, and the bottom two layers of Pd atoms were fixed during structural geometry optimization.

The adsorption energy was calculated using the equation, $E_{ads} = E_{x/slab} – (E_{slab} + E_x)$, where $E_{x/slab}$ is the total energy of the slab with adsorbates in its equilibrium geometry, E_{slab} is the energy of the bare slab, and E_x is the energy of the free adsorbates in the gas phase. Therefore, the more negative the E_{ads}, the stronger the adsorption. The reaction enthalpies (ΔE) were calculated using the equation, $\Delta E = E_{FS} – E_{IS}$, where E_{IS} and E_{FS} are the total energies of the corresponding initial state (E_{IS}) and final state (E_{FS}).

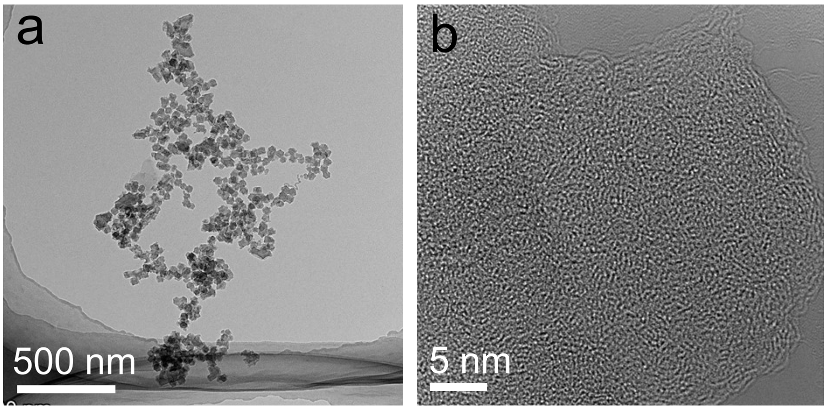

Figure S1. TEM images of FeNC.

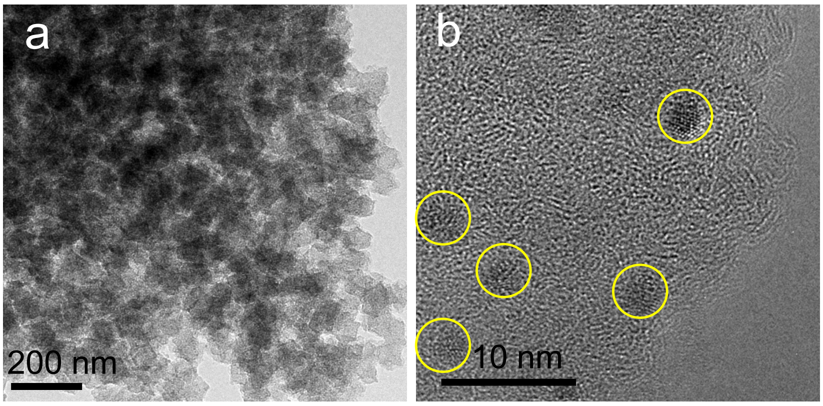

Figure S2. TEM images of FeNC-Pd_{NC}.

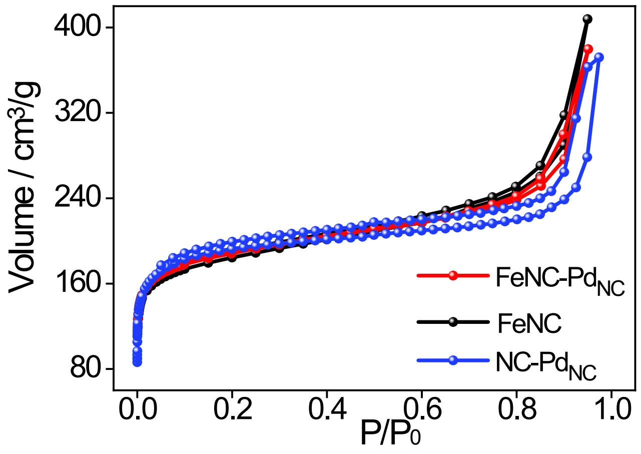

Figure S3. N_2 adsorption/desorption isotherms of FeNC-Pd_{NC}, FeNC and NC-Pd_{NC}.

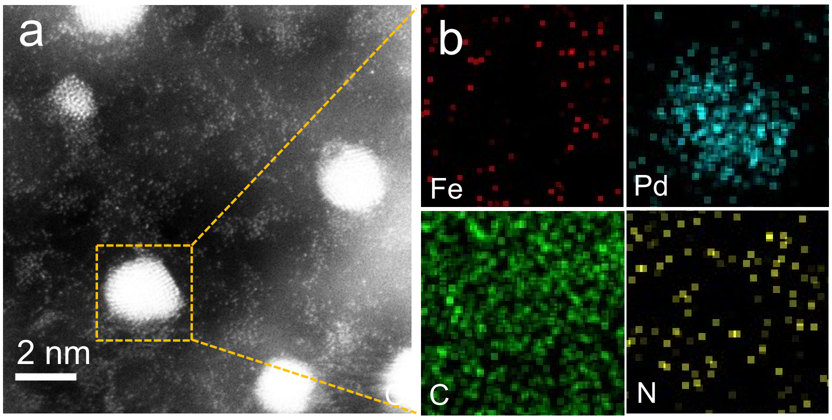

Figure S4. (a) AC-HAADF STEM image and (b) EDS elemental (Fe, Pd, C, and N) mapping analysis of FeNC-Pd_{NC}.

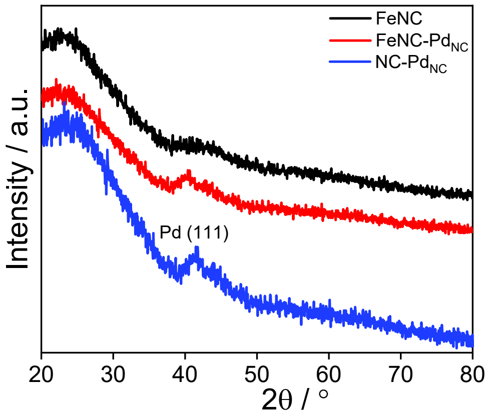

Figure S5. XRD patterns of FeNC-Pd_{NC}, FeNC, and NC-Pd_{NC}.

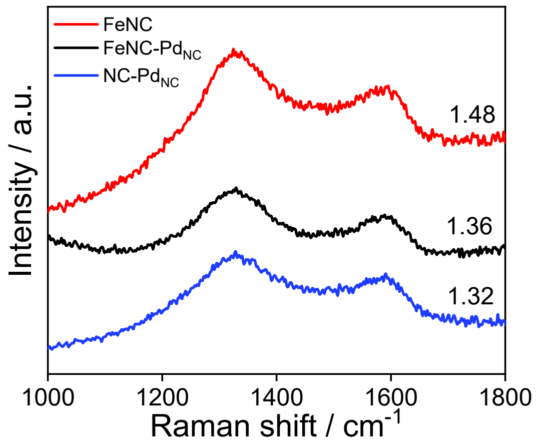

Figure S6. Raman spectra of FeNC-Pd$_{NC}$, FeNC, and NC-Pd$_{NC}$. The ratio of *D* and *G* band intensities are shown inside the figure.

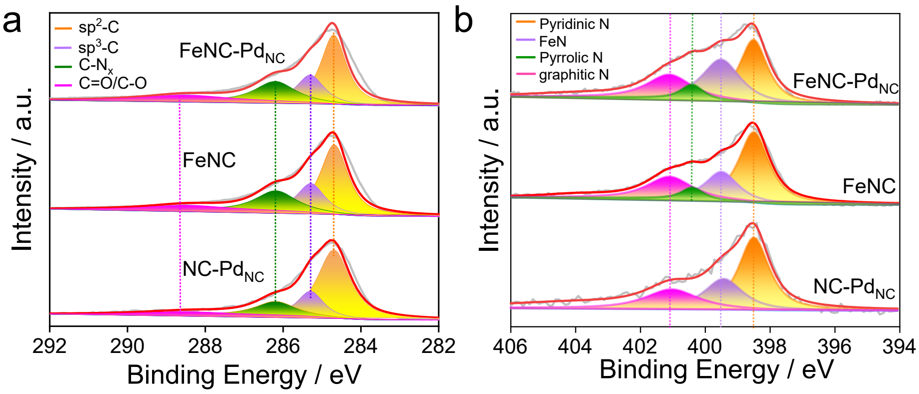

Figure S7. (a) High-resolution C 1s and (b) N 1s XPS spectra of FeNC-Pd$_{NC}$, FeNC, and NC-Pd$_{NC}$.

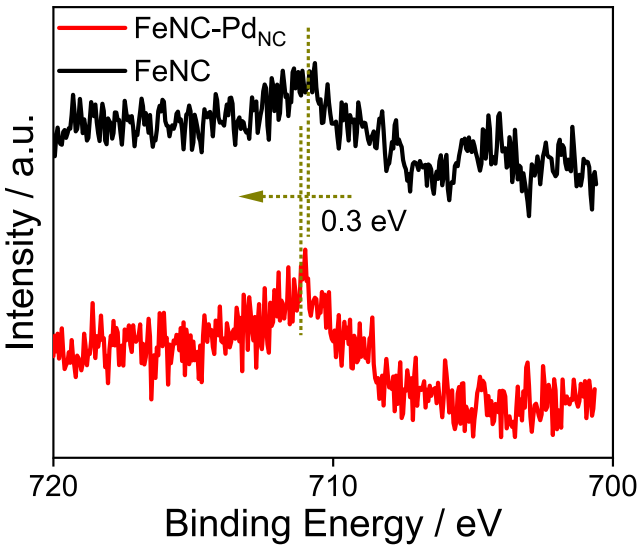

Figure S8. High-resolution Fe 2d XPS spectra of FeNC-Pd$_{NC}$ and FeNC.

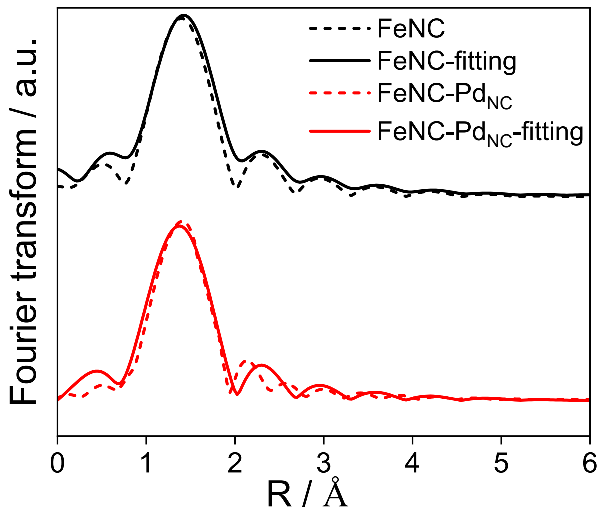

Figure S9. EXAFS spectra fitting results of FeNC-Pd$_{NC}$ and FeNC.

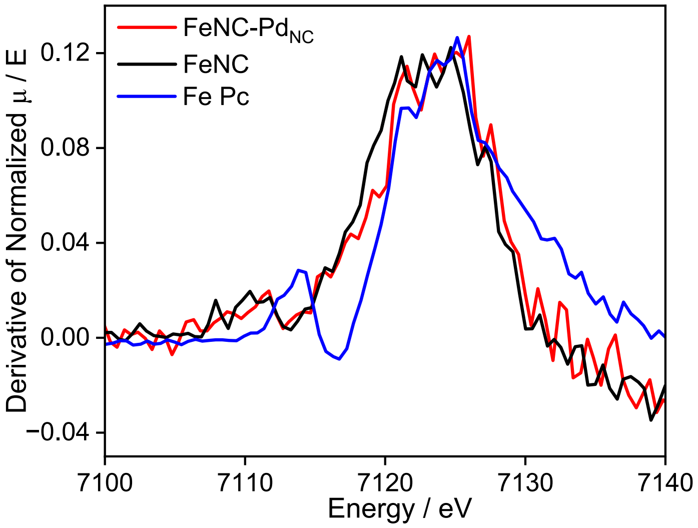

Figure S10. First derivative of XANES spectra of FeNC-Pd$_{NC}$, FeNC, and Fe Pc.

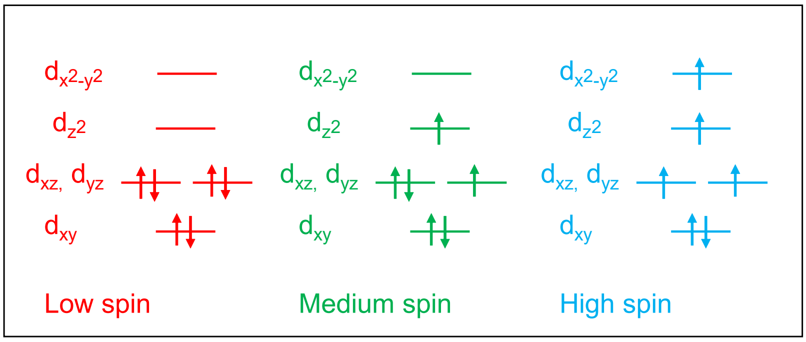

Figure S11. Schematic of the different spin states of Fe(II) atom.

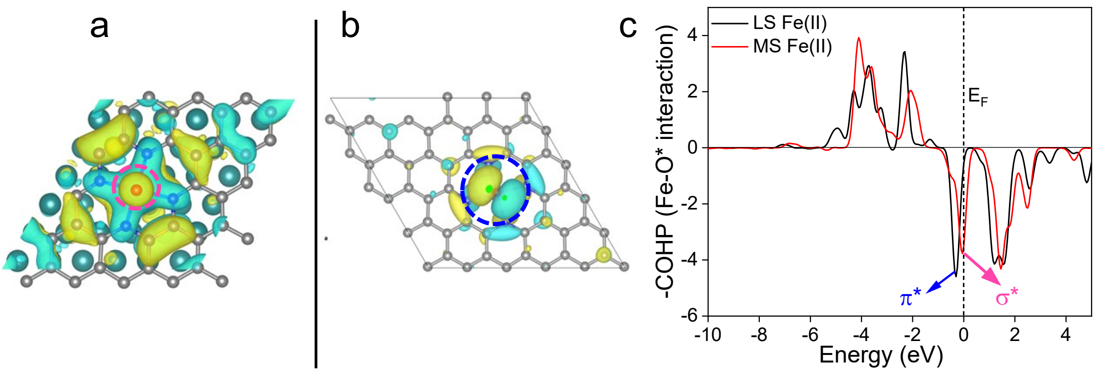

Figure S12. Highest occupied molecular orbital of O* adsorbed (a) low-spin (LS) Fe(II) atom and (b) medium-spin (MS) Fe(II) atom. The yellow and cyan regions represent electron accumulation and depletion, respectively. (c) Calculated projected crystal orbital Hamilton population (pCOHP) diagram of adsorbed *O on both LS Fe(II) and MS Fe(II) atoms.

Note for Figure S12: An additional σ* bond is produced between the partially occupied MS Fe(II) and the π orbital of the O* compared with FeNC (Figure S12a-b). Owing to this σ* contribution, the projected crystal orbital Hamilton population (pCOHP) analysis of Fe–O interaction in O* adsorption intermediates shows that the integrated COHP (|ICOHP|) value of MS Fe(II) (2.58) is smaller than LS Fe(II) (2.75), which suggest weak Fe–O* bond strength (Figures S12c).

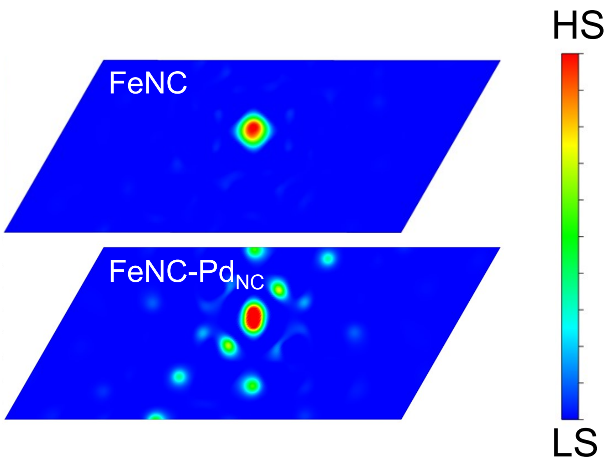

Figure S13. 2D spin density diagrams of FeNC-Pd$_{NC}$ (top) and Fe-N-C (bottom). The isosurface is 0.1 a.u.

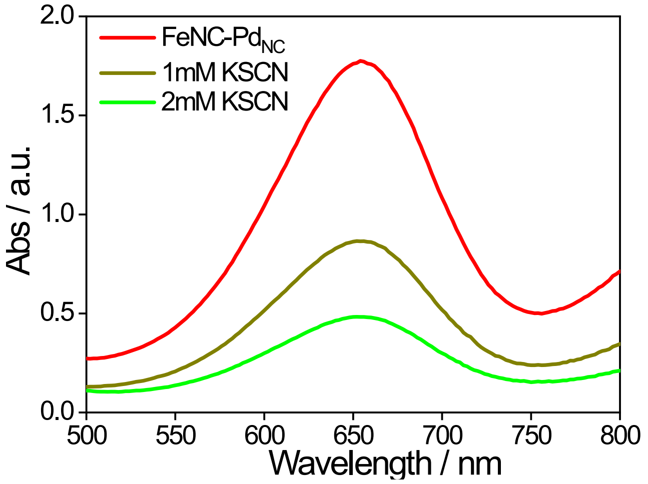

Figure S14. UV-vis spectra of FeNC-Pd$_{NC}$ before and after treatment with KSCN in the TMB/ H_2O_2 system.

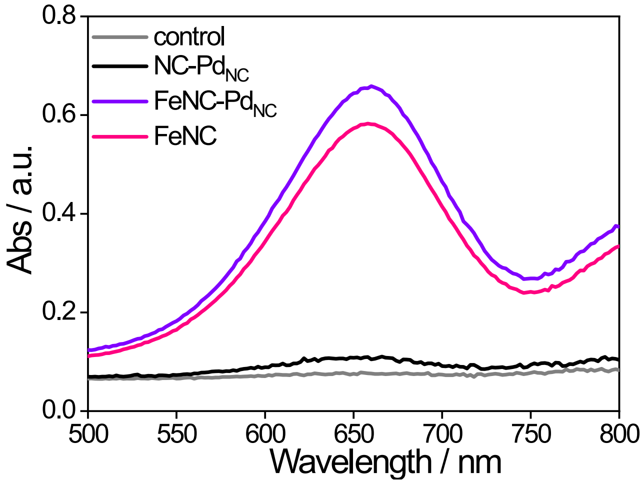

Figure S15. UV-vis spectra of FeNC-Pd$_{NC}$, FeNC, and NC-Pd$_{NC}$ in the TMB system.

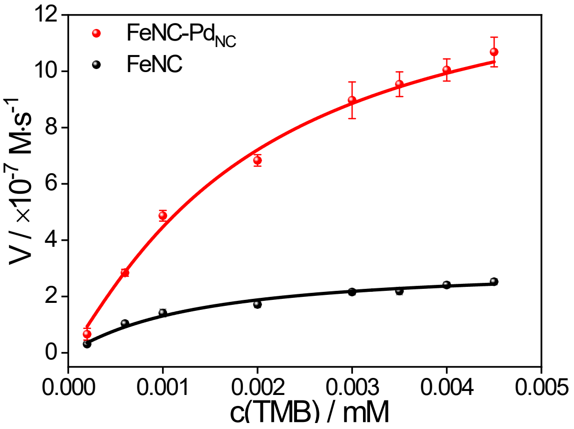

Figure S16. Michaelis-Menten kinetic analysis of FeNC-Pd_{NC} and FeNC using different concentrations of TMB.

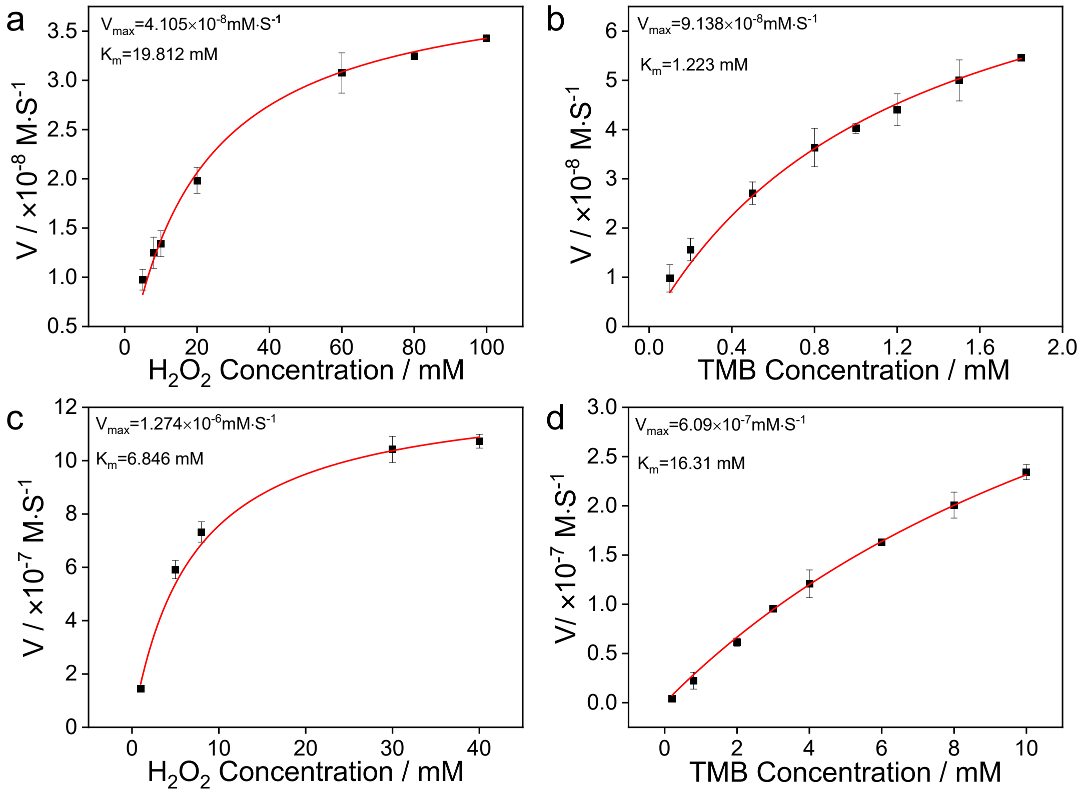

Figure S17. Michaelis–Menten kinetic analysis of NC-Pd_{NC} using different concentrations of (a) H_2O_2 and (b) TMB. Michaelis–Menten kinetic analysis of HRP using different concentrations of (c) H_2O_2 and (d) TMB.

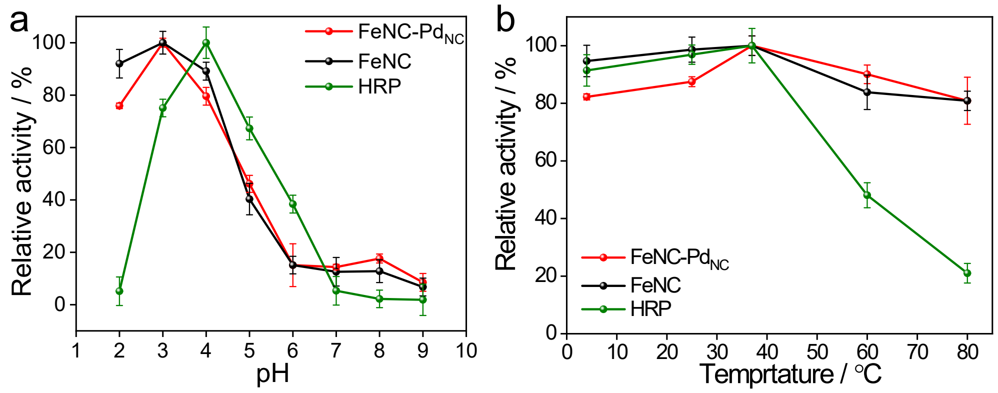

Figure S18. Influence of (a) pH and (b) temperatures on the POD-like activity of FeNC-Pd$_{NC}$, FeNC, and HRP.

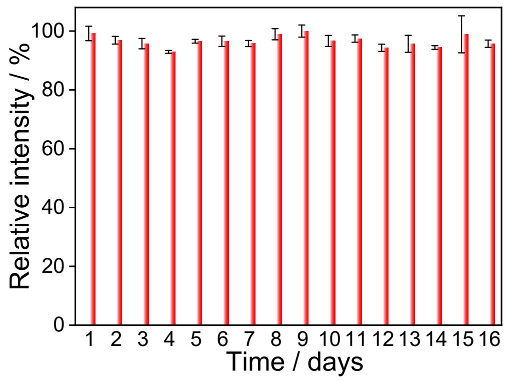

Figure S19. Long-term stability of POD-like activity of FeNC-Pd$_{NC}$.

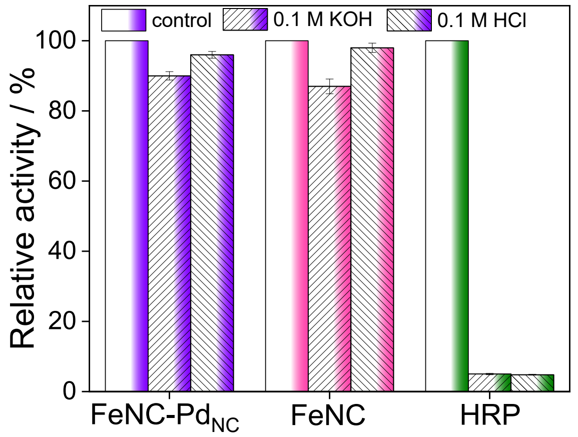

Figure S20. Relative activities of FeNC-Pd$_{NC}$, FeNC, and HRP in 0.1 M KOH and 0.1 M HCl solutions.

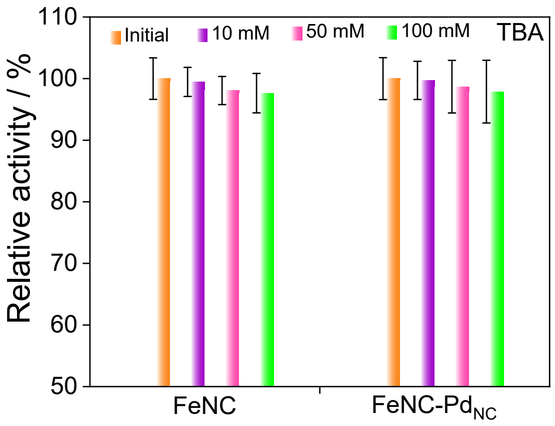

Figure S21. Effect of t-butyl alcohol (TBA) on the POD-like activity of FeNC-Pd$_{NC}$ and FeNC.

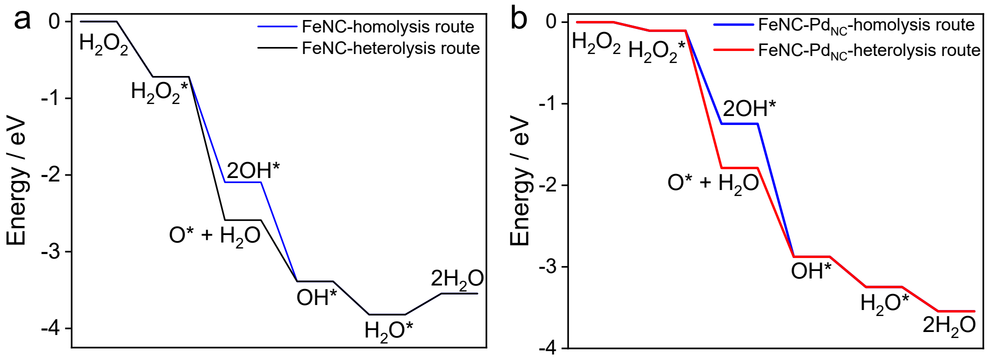

Figure S22. Free-energy diagrams of the homolysis and heterolysis mechanisms of the POD-like reaction pathways occurring on (a) FeNC and (b) FeNC-Pd$_{NC}$.

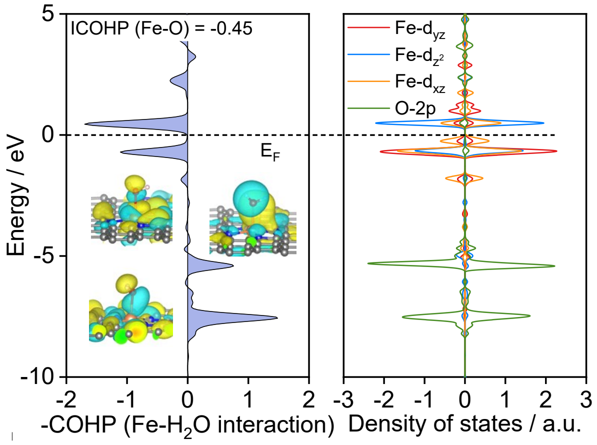

Figure S23. Calculated pCOHP and density of states diagram of H_2O* on FeNC.

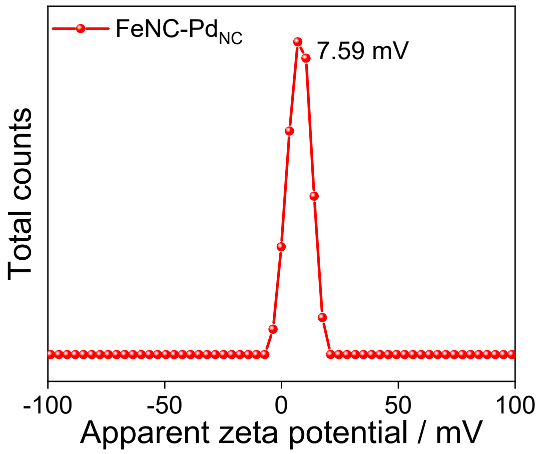

Figure S24. Zeta potential of FeNC-Pd_{NC}.

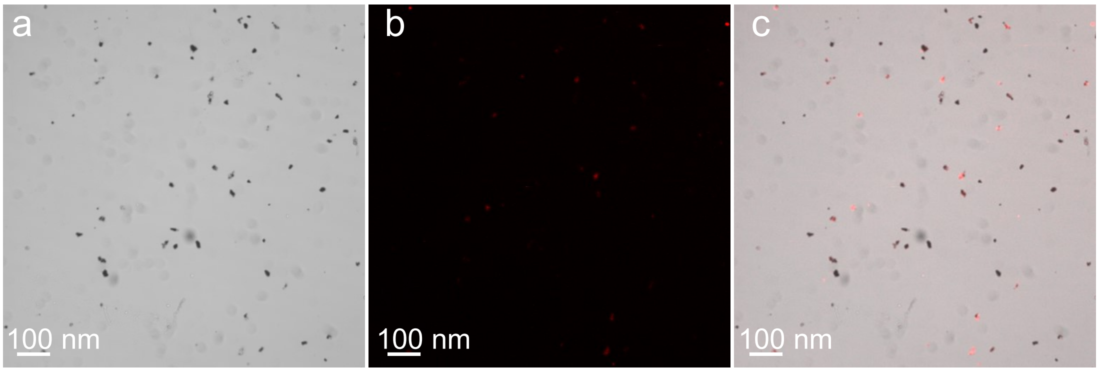

Figure S25. (a) Light field image and (b) fluorescence image at 488 nm of FeNC-Pd_{NC}/Ab_2 and (c) merged image of both (a) and (b).

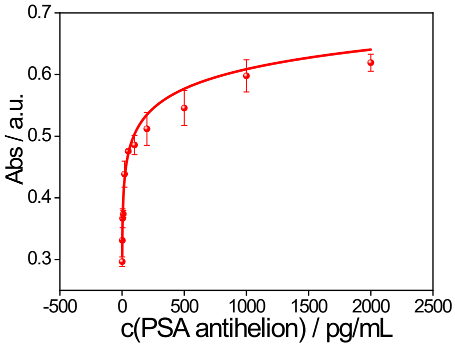

Figure S26. Calibration curve of the absorbance intensity of ox-TMB and concentration of PSA for the FeNC-Pd_{NC} assay. Each data point is the mean of triplicate readings.

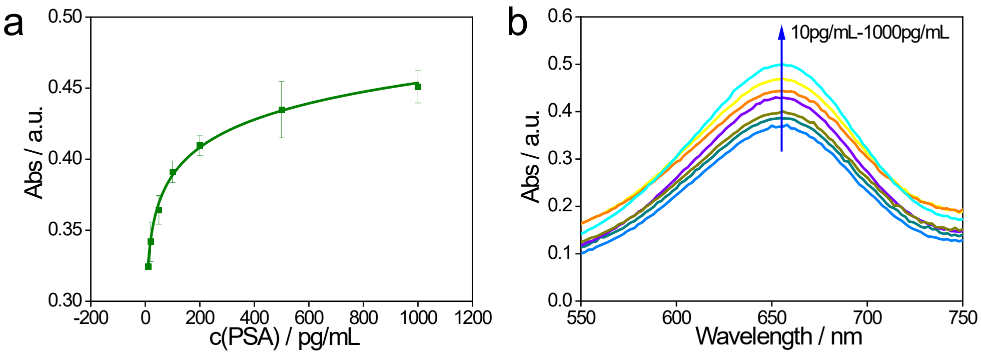

Figure S27. (a) Calibration curve of the absorbance intensity of ox-TMB and concentration of PSA for the HRP ELISA. Each data point is the mean of triplicate readings**. (b) Absorption spectra of ox-TMB with different PSA concentrations using HRP as the lable.**

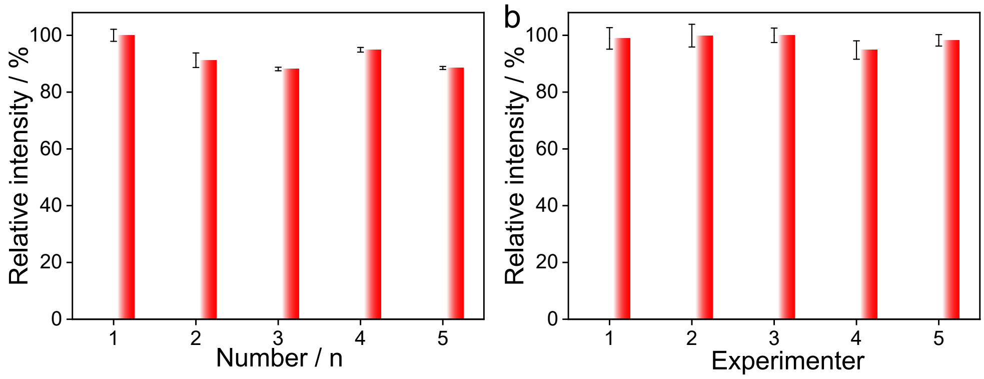

Figure S28. (a) Repeatability and (b) reproducibility of FeNC-Pd_{NC} NLISA for the determination of PSA.

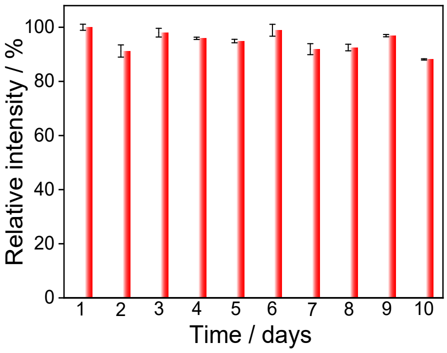

Figure S29. Stability of FeNC-Pd_{NC} NLISA during the detection of PSA.

Table S1. BET surface area of FeNC-Pd_{NC}, FeNC, and NC-Pd_{NC}

Nanozymes	BET Surface area (m^2/g)
FeNC-Pd_{NC}	547.0
FeNC	554.9
NC-Pd_{NC}	550.5

Table S2. XPS results of FeNC-Pd_{NC}, FeNC, and NC-Pd_{NC}

Nanozymes		FeNC-Pd_{NC}	FeNC	NC-Pd_{NC}
Relative amounts of various elements (%)	Fe	0.4	0.4	-
	Pd	4.2	-	4.6
	C	83.2	85.5	85.1
	N	8.3	8.2	8.3
	O	3.9	6.0	16.3
Relative amounts of C species (%)	sp^2-C	46.3	49.3	60.7
	sp^3-C	19.7	20.6	17.8
	C-N	21.4	21.1	15.1
	O-C=O	12.6	8.9	6.3
Relative amounts of N species (%)	Pyridinic N	47.6	49.6	57.7
	FeN	23.1	21.6	-
	Pyrrolic N	7.1	6.3	10.3
	Graphitic N	22.2	22.5	32.0

Table S3. EXAFS fitting parameters at the Fe K-edge of FeNC-Pd_{NC} and FeNC(S_0^2=0.9).

Nanozymes s	N	R(Å)	σ^2(Å^2)	ΔE_0(eV)	R factor
FeNC-Pd_{NC}	4.1	1.94	0.0018	-6.6	1.94×10^{-3}
FeNC	4.2	1.93	0.0080	-2.8	1.87×10^{-3}

S_0^2: amplitude reduction factor; N: coordination numbers; R: bond distance; σ^2: Debye–Waller factors; ΔE_0: inner potential correction; R factor: goodness of fit.

Table S4. Room temperature ^{57}Fe Mössbauer parameters for ^{57}Fe-enriched FeNC-Pd$_{NC}$ and FeNC

Catalysts	Fe species	Assignment	IS/ mm·s^{-1}	QS/ mm·s^{-1}	Line width/ mm·s^{-1}	Content / %
Fe-N-C/Pd$_{NC}$	D1	LS	0.35	0.82	0.58	50.4 %
	D2	MS	0.11	2.08	0.58	33.4 %
	D3	HS	0.63	2.02	0.58	16.3 %
Fe-N-C	D1	LS	0.38	0.80	0.58	70.3 %
	D2	MS	0.12	2.41	0.58	17.1 %
	D3	HS	0.78	2.33	0.58	12.6 %

Table S5. Enzyme kinetics data of FeNC-Pd_{NC} and FeNC.

POD mimics	S (μM)	Substrate	K_m (mM)	V_{max} (μM s^{-1})	K_{cat} (s^{-1})	References
FeNC-Pd_{NC}	2.14	H_2O_2	9.58	10.93	5.10	This work
		TMB	1.97	14.30	6.68	
FeNC	2.14	H_2O_2	14.02	5.49	2.56	
		TMB	1.35	3.10	1.45	
NC-Pd_{NC}	17.5	H_2O_2	19.81	0.041	0.0023	
		TMB	1.22	0.091	0.0052	
Zn-N-C	48	H_2O_2	40.16	1.251	0.0367	[4]
		TMB	0.224	0056	0.0022	
Fe-N-C	7.48	H_2O_2	24.25	5.48	0.073	[5]
		TMB	1.48	5.6	0.075	
NG-Heme	2.97	H_2O_2	0.0167	0.0602	0.056	[6]
		TMB	0.0602	1.67	0.2	
Fe-MOF	49	H_2O_2	1.3	5.6	0.0005	[7]
		TMB	2.6	6.2	0.0011	
Cu-N-C	2.68	H_2O_2	19.94	0.2	0.075	[8]
		TMB	3.76	0.75	0.283	
Pt hollow nanodendrites	7	H_2O_2	6.9	0.099	0.014	[9]
		TMB	0.81	0.12	0.0069	
HRP	2.27×10^{-5}	H_2O_2	6.85	1.27	5.6×10^{4}	This work
		TMB	16.31	0.61	2.7×10^{4}	

S is the concentration of metal atom, which is obtained from ICP-OES. The Fe content in FeNC-Pd_{NC} and FeNC was 0.36 wt%, while the Pd content in FeNC-Pd_{NC} was 5.6 wt%.

Table S6. Comparison of various materials and methods used in PSA biosensors

**Detection system**	**Methods**	**Linear range /ng/mL**	**Detection limit /pg/mL**	**RSD/%**	**Specificity**	**Ref.**
PF-TC6FQ Pdots	Fluorescence	2-10	32	-	CEA, AFP, FBS, HSA	[10]
CDs	Fluorescence	0.1-100	22	1.2-6.7	HSA, Hb, IgG, CEA	[11]
crumpled GR-Au	CV	0-10	59	3.1	AFP, UA, BSA, Glu, VitC	[12]
AuNPs-PAMAM	EIS	0.05-35	5	3.2	BSA, AFP	[13]
FPZ-ELISA	Colorimetric	8-1000	1.8	-	CEA, BSA, ER, HER2, PR	[14]
SPCE/GO-CO2H/apta/cDNA/MB	DPV	0.001-100	0.064	-	IgG, CEA, HSA	[15]
MIP/PTBs/Au electrode	DPV	1-60	1	1.86-1.94	CEA, HSA	[16]
GO-AgNPs nanocomposite	LSV	0.005-20	0.33		CEA, AFP, BSA	[17]
Au/apta-MIP	Capacitance	0.1-100	1	-	HK2, HSA	[18]
SERS dots/Ab1/PSA/Ab2/SERS dots	SERS	0.001-1000	0.11	-	-	[19]
anti-PSA/MCH/AgNPs/SiNWs	SERS	0.1-20	0.1	5.0-10.01	BSA, HSA	[20]
GO-AgNPs	Raman spectroscopy	0.0005-0.5	0.23	5.95-14.38	AFP, CEA, GSH, Lys, Ala, ALP, HSA, Glb	[21]
DNA-AgNC	Fluorescence	2-150	1140	-	AFP, CEA, HCG, HIgG	[22]

PtCu HNFs	DPV	0.01-100	3	4.31	CA-125, CEA, AFP, BSA	[14]
AuNPs/rGO/THI	DPV	0.05-200	10	8.84	CEA, NSE, AA, BSA, IgG	[23]
GO/Fe_3O_4-DNA	Colorimetric/photothermal	1-128	310	-	BSA, HGB, CEA	[24]
CdS@NiS nanocomposites	Photoelectrochemistry	0.01-50	2.9	9.67	PSA, CEA, AFP, IgG	[25]
FAM	Fluorescence	0.001-0.1	0.76	3.13-6.39	HSA, AFP, CEA	[26]
DNA origami	Fluorescence	0.2-3	30	3.2-7.6	HSA, IgG, CEA, AFP	[27]
FeNC-Pd_{NC}- ELISA	Colorimetric	1-2000	0.38	0.18-5.18	BSA, CEA, ESR1, HER2, CAT, GO_x, Zn^{2+}, K^+, Na^+	This work

CV: cyclic voltammetry; EIS: electrochemical impedance spectroscopy; DPV: differential pulse voltammetry; LSV: linear sweep voltammetry; SERS: surface-enhanced Raman scattering.
CEA: carcinoembryonic antigen; AFP: alpha-fetoprotein; FBS: fetal bovine serum; HSA: human serum albumin; IgG: human immunoglobulin G; UA: uric acid; BSA: bovine serum albumin; Glu: glucose; Vit C: vitamin C; HK2: human glandular kallikrein 2; GSH: glutathione; Lys: lysine; Ala: alanine; ALP: alkaline phosphatase; Glb: globulin; HCG: human chorionic gonadotropin; HigG: human immunoglobulin; CA-125: cancer antigen 125; NSE: neuron-specific enolase ; AA: ascorbic acid; ESR1: estrogen receptor alpha; HER2: human epidermal growth factor receptor-2; GO_x: glucose oxidase.

Supplementary References

1. Kresse, G.; Hafner, J., Ab Initio Molecular Dynamics for Liquid Metals. *Phys. Rev. B Condens. Matter.* **1993,** *47*, 558-561.

2. Perdew, J.; Burke, K.; Ernzerhof, M. Generalized Gradient Approximation Made Simple. *Phys. Rev. Lett.* **1997**, *78*, 1396.

3. Gong, L.; Zhang, H.; Wang, Y.; Luo, E.; Li, K.; Gao, L.; Wang, Y.; Wu, Z.; Jin, Z.; Ge, J.; Jiang, Z.; Liu, C.; Xing, W., Bridge Bonded Oxygen Ligands Between Approximated FeN4 Sites Confer Catalysts with High ORR Performance. *Angew. Chem. Int. Ed.* **2020**, *59*, 13923-13928.

4. Xu, B.; Wang, H.; Wang, W.; Gao, L.; Li, S.; Pan, X.; Wang, H.; Yang, H.; Meng, X.; Wu, Q.; Zheng, L.; Chen, S.; Shi, X.; Fan, K.; Yan, X.; Liu, H., A Single-Atom Nanozyme for Wound Disinfection Applications. *Angew. Chem. Int. Ed.* **2019**, *58*, 4911-4916.

5. Jiao, L.; Xu, W.; Zhang, Y.; Wu, Y.; Gu, W.; Ge, X.; Chen, B.; Zhu, C.; Guo, S., Boron-Doped Fe-N-C Single-Atom Nanozymes Specifically Boost Peroxidase-Like Activity. *Nano Today* **2020**, *35*.

6. Xu, W.; Song, W.; Kang, Y.; Jiao, L.; Wu, Y.; Chen, Y.; Cai, X.; Zheng, L.; Gu, W.; Zhu, C., Axial Ligand-Engineered Single-Atom Catalysts with Boosted Enzyme-Like Activity for Sensitive Immunoassay. *Anal. Chem.* **2021**, *93*, 12758-12766.

7. Xu, W.; Jiao, L.; Yan, H.; Wu, Y.; Chen, L.; Gu, W.; Du, D.; Lin, Y.; Zhu, C., Glucose Oxidase-Integrated Metal-Organic Framework Hybrids as Biomimetic Cascade Nanozymes for Ultrasensitive Glucose Biosensing. *ACS Appl. Mater. Interfaces* **2019**, *11*, 22096-22101.

8. Wu, Y.; Wu, J.; Jiao, L.; Xu, W.; Wang, H.; Wei, X.; Gu, W.; Ren, G.; Zhang, N.; Zhang, Q.; Huang, L.; Gu, L.; Zhu, C., Cascade Reaction System Integrating Single-Atom Nanozymes with Abundant Cu Sites for Enhanced Biosensing. *Anal. Chem.* **2020**, *92*, 3373-3379.

9. Ge, C.; Wu, R.; Chong, Y.; Fang, G.; Jiang, X.; Pan, Y.; Chen, C.; Yin, J.-J., Synthesis of Pt Hollow Nanodendrites with Enhanced Peroxidase-Like Activity Against Bacterial Infections: Implication for Wound Healing. *Adv. Funct. Mater.* **2018**, *28*, 1801484.

10. Yang, Y. Q.; Yang, Y. C.; Liu, M. H.; Chan, Y. H., FRET-Created Traffic Light Immunoassay Based on Polymer Dots for PSA Detection. *Anal. Chem.* **2019**, *92*, 1493-1501.

11. He, J. H.; Cheng, Y. Y.; Zhang, Q. Q.; Liu, H.; Huang, C. Z., Carbon Dots-Based Fluorescence Resonance Energy Transfer for the Prostate Specific Antigen (PSA) with High Sensitivity. *Talanta* **2020,** *219*, 121276.

12. Jang, H. D.; Kim, S. K.; Chang, H.; Choi, J. W., 3D Label-Free Prostate Specific Antigen (PSA) Immunosensor Based on Graphene-Gold Composites. *Biosens. Bioelectron.* **2015,** *63*, 546-551.

13. Kavosi, B.; Salimi, A.; Hallaj, R.; Moradi, F., Ultrasensitive Electrochemical Immunosensor for PSA Biomarker Detection in Prostate Cancer Cells Using Gold Nanoparticles/PAMAM Dendrimer Loaded with Enzyme Linked Aptamer as Integrated Triple Signal Amplification Strategy. *Biosens. Bioelectron.* **2015,** *74*, 915-923.

14. Chen, Y.; Jiao, L.; Yan, H.; Xu, W.; Wu, Y.; Zheng, L.; Gu, W.; Zhu, C., Fe-N-C Single-Atom Catalyst Coupling with Pt Clusters Boosts Peroxidase-like Activity for Cascade-Amplified Colorimetric Immunoassay. *Anal. Chem.* **2021,** *93*, 12353-12359.

15. Raouafi, A.; Sánchez, A.; Raouafi, N.;Villalonga, R., Electrochemical Aptamer-Based Bioplatform for Ultrasensitive Detection of Prostate Specific Antigen. *Sens. Actuators B Chem.* **2019,** *297*, 126762.
16. Abbasy, L.; Mohammadzadeh, A.; Hasanzadeh, M.; Razmi, N., Development of a Reliable Bioanalytical Method Based on Prostate Specific Antigen Trapping on the Cavity of Molecular Imprinted Polymer Towards Sensing of PSA Using Binding Affinity of PSA-MIP Receptor: A Novel Biosensor. *J. Pharm. Biomed. Anal.* **2020,** *188*, 113447.
17. Meng, F.; Sun, H.; Huang, Y.; Tang, Y.; Chen, Q.; Miao, P., Peptide Cleavage-Based Electrochemical Biosensor Coupling Graphene Oxide and Silver Nanoparticles. *Anal. Chim. Acta.* **2019,** *1047*, 45-51.
18. Jolly, P.; Tamboli, V.; Harniman, R. L.; Estrela, P.; Allender, C. J.; Bowen, J. L., Aptamer-MIP Hybrid Receptor for Highly Sensitive Electrochemical Detection of Prostate Specific Antigen. *Biosens. Bioelectron.* **2016,** *75*, 188-95.
19. Chang, H.; Kang, H.; Ko, E.; Jun, B.-H.; Lee, H.-Y.; Lee, Y.-S.; Jeong, D. H., PSA Detection with Femtomolar Sensitivity and a Broad Dynamic Range Using SERS Nanoprobes and an Area-Scanning Method. *ACS Sens.* **2016,** *1*, 645-649.
20. Ouhibi, A.; Raouafi, A.; Lorrain, N.; Guendouz, M.; Raouafi, N.; Moadhen, A., Functionalized SERS Substrate Based on Silicon Nanowires for Rapid Detection of Prostate Specific Antigen. *Sens. Actuators B Chem.* **2021,** *330,* 129352.
21. Yang, L.; Zhen, S. J.; Li, Y. F.; Huang, C. Z., Silver Nanoparticles Deposited on Graphene Oxide for Ultrasensitive Surface-Enhanced Raman Scattering Immunoassay of Cancer Biomarker. *Nanoscale* **2018,** *10*, 11942-11947.
22. Fang, B. Y.; An, J.; Liu, B.; Zhao, Y. D., Hybridization Induced Fluorescence Enhanced DNA-Ag Nanocluster/Aptamer Probe for Detection of Prostate-Specific Antigen. *Colloids Surf. B Biointerfaces* **2019,** *175*, 358-364.
23. Wei, B.; Mao, K.; Liu, N.; Zhang, M.; Yang, Z., Graphene Nanocomposites Modified Electrochemical Aptamer Sensor for Rapid and Highly Sensitive Detection of Prostate Specific Antigen. *Biosens. Bioelectron.* **2018,** *121*, 41-46.
24. Wei, Y.; Wang, D.; Zhang, Y.; Sui, J.; Xu, Z., Multicolor and Photothermal Dual-Readout Biosensor for Visual Detection of Prostate Specific Antigen. *Biosens Bioelectron* **2019,** *140*, 111345.
25. Zhu, L.; Lv, Z.; Yin, Z.; Tang, D., Double Ion-Exchange Reaction-Based Photoelectrochemical Immunoassay for Sensitive Detection of Prostate-Specific Antigen. *Anal. Chim. Acta.* **2021,** *1149*, 338215.
26. Yan, Y.; Ma, C.; Tang, Z.; Chen, M.; Zhao, H., A Novel Fluorescent Assay Based on DNAzyme-Assisted Detection of Prostate Specific Antigen for Signal Amplification. *Anal. Chim. Acta.* **2020,** *1104*, 172-179.
27. Taghdisi, S. M.; Danesh, N. M.; Nameghi, M. A.; Ramezani, M.; Alibolandi, M.; Abnous, K., A DNA Triangular Prism-Based Fluorescent Aptasensor for Ultrasensitive Detection of Prostate-Specific Antigen. *Anal. Chim. Acta.* **2020,** *1120*, 36-42.
